# To be or Not to be Hybrids? A Case‐Study in an Allochthonous Population Involving Illegal Introductions of the Endangered Golden‐Headed and Golden Lion Tamarins

**DOI:** 10.1002/ajp.70112

**Published:** 2025-12-31

**Authors:** Gabriela Aliaga‐Samanez, Carla Gestich, Paola Andrea Ayala‐Burbano, Nathalia Bulhões Javarotti, Katherine McVay, Maria Cecilia Martins Kierulff, Ana Paula Cazerta Farro, Sílvia Bahadian Moreira, Alcides Pissinatti, Amy Goldberg, Pedro M. Galetti, Patrícia Domingues de Freitas

**Affiliations:** ^1^ Departamento de Genética e Evolução Universidade Federal de São Carlos São Carlos Brazil; ^2^ Department of Wildlife Diseases Leibniz Institute for Zoo and Wildlife Research Berlin Germany; ^3^ Grupo de investigación GIESUM, Facultad de Ciencias de la Salud, Universidad Mariana, San Juan de Pasto Nariño Colombia; ^4^ Department of Human Genetics University of California Los Angeles California USA; ^5^ Laboratório de Genética e Conservação Animal, Centro Universitário Norte do Espírito Santo, Universidade Federal do Espírito Santo (UFES) São Mateus Brazil; ^6^ Centro de Primatologia do Rio de Janeiro (CPRJ‐INEA) Guapimirim Brazil

**Keywords:** alien species, conservation genetics, genotyping by sequencing, Leontopithecus

## Abstract

Allochthonous species can negatively impact biodiversity by introducing new pathogens, intensifying competition for resources, promoting habitat changes, and causing ecosystem disruption. Introduced species can also spread into areas designated for the conservation of native species. This colonization and, eventually, contact between historically isolated lineages can cause hybridization, resulting in decreased offspring fitness or, conversely, promoting hybrid vigor, threatening the integrity of native populations and increasing their risk of extinction. Here, we analyzed an allochthonous population from a fragment of Brazilian Atlantic Forest, where both endangered Golden‐headed lion tamarins and Golden lion tamarins were illegally introduced into the natural range of the Golden lion tamarin in the 1990s. We performed Genotyping by Sequencing and complementary mitochondrial analysis to investigate hybridization and to assess the genetic diversity and structure of the invasive population. Our results were able to rule out the existence of hybrids among the samples analyzed, providing relevant information about the genetic diversity of the alien population and effectively contributing to conservation programs and management actions for both species. Conservation actions and effective surveillance policies to prevent new illegal introductions should be mandatory to ensure the protection of these threatened charismatic species which are targets of illegal trade and trafficking.

## Graphical abstract

Genetic analyses of an allochthonous population of *Leontopithecus*, illegally introduced into an area of the Brazilian Atlantic Forest in Rio de Janeiro, did not detect signs of hybridization. This data provides crucial information to support conservation efforts and prevent new threats to the endangered lion tamarins.

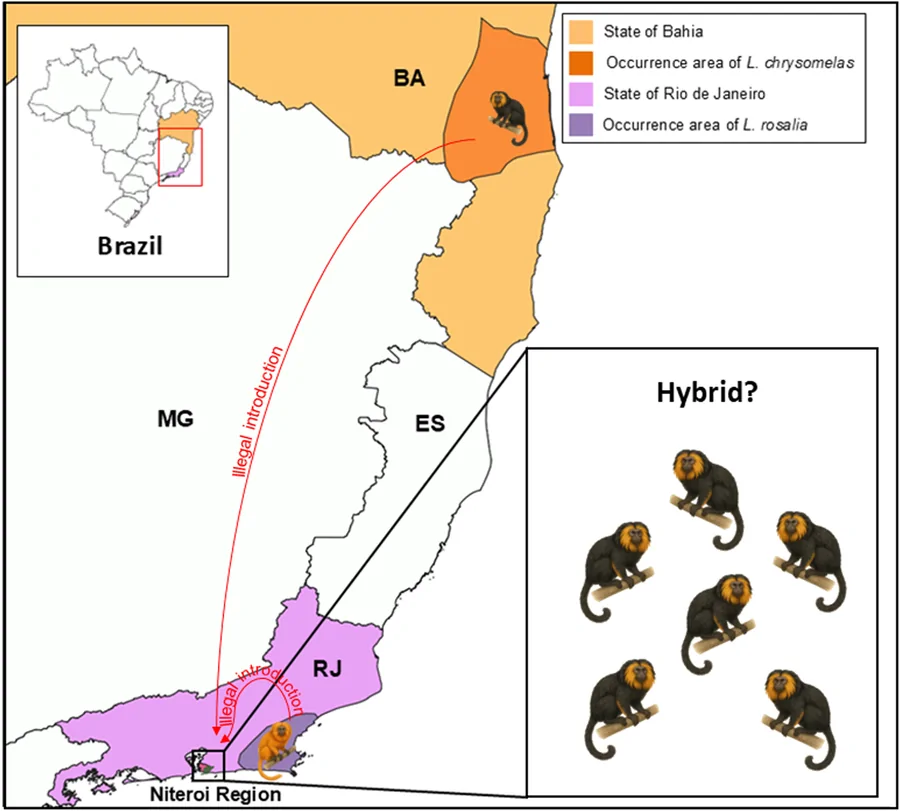

## 1 Introduction

The translocation of an organism from one area to another, within its historical range, can serve as a valuable tool for conservation purposes (IUCN/SSC 2013). However, translocation can have negative impacts on local species and even lead to biodiversity loss when it occurs in an area never previously occupied by the translocated species, constituting the introduction of an alien species (Walker and Steffen 1997). Translocation of alien individuals may increase competition for resources (Blanchet et al. 2007; Ruiz‐Miranda et al. 2006), introduce new diseases to the environment (Mack and D'Antonio 1998), cause habitat changes and ecosystem disruptions (Ellstrand and Schierenbeck 2000), and/or promote undesired hybridization with phylogenetically related species (Todesco et al. 2016).

Hybridization is known to produce offspring with reduced fitness, potentially leading to the extinction of hybrid individuals (Allendorf et al. 2001, 2013). However, it can also enhance genetic diversity, fostering the development and emergence of new lineages. Moreover, hybrids can exhibit heterosis or hybrid *vigor*, which may lead to their superiority over non‐hybrid species (Jaquish 1994; Lippman and Zamir 2007; Smith and Scott 1989). Fertile hybrids can negatively impact the survival and maintenance of pure species or parental lineages (Cortés‐Ortiz 2017; Park 2004; Rhymer and Simberloff 1996), increasing the risk of extinction, especially of threatened or rare taxa. This scenario can be even more harmful to local biodiversity if promoted by illegal human activities, without legal authorization or adequate monitoring, mainly affecting species with some degree of threat, such as lion tamarins (SALVE 2025).

Lion tamarins are Neotropical primates of the genus *Leontopithecus* (Callitrichidae, Platyrrhini), which comprises four allopatric species endemic to the Brazilian Atlantic Forest (Mundy and Kelly 2001). The Black‐faced Lion Tamarin (BFLT, *Leontopithecus caissara*) is found exclusively in a narrow area of southeastern coastal Atlantic Forest at the border of the states of Parana and São Paulo (Kierulff and Rylands 2008a), while the Black Lion Tamarin (BLT, *Leontopithecus chrysopygus*) is endemic to the inland Atlantic Forest of São Paulo state (Kierulff and Rylands 2008c). The Golden Lion Tamarin (GLT, *Leontopithecus rosalia*) inhabits the coastal Atlantic Forest in the state of Rio de Janeiro (Ruiz‐Miranda et al. 2019), and the Golden‐headed Lion Tamarin (GHLT, *Leontopithecus chrysomelas*) is endemic to the lowland Atlantic Forest in the south of the Bahia state (Kierulff and Rylands 2008b). All four species face some level of threat, as classified by the International Union for Conservation of Nature Red List (IUCN 2025) and the Red Book of the Brazilian Environmental Ministry and Climate Change (MMA 2022).

The conservation of the lion tamarins has currently been considered a major challenge, mainly due to the negative impacts caused by the disordered human demographic growth, irresponsible use of natural resources (Coimbra‐Filho 2004), deforestation, habitat loss and fragmentation, in addition to hunting and illegal trade (Caballero and R 2014). According to the Pri‐Matas Institute, a non‐governmental organization responsible for the *in‐situ* management of invasive lion tamarins, in 1994 there was an illegal release of individuals of *L. chrysomelas*, along with few *L. rosalia* individuals, in an area adjacent to the current natural occurrence of the *L. rosalia* (unpublished data). The illegal introduction or release of allochthonous species into natural distribution range areas of closely‐related species had already been reported for other Neotropical species (Oliveira and Grelle 2012), causing concern about how an exotic species can affect local biodiversity, by favoring its spread and population growth, becoming an invasive species (Seebens et al. 2017), or/and by the emergence of artificial hybridization zones, where reproduction can produce viable and fertile progeny (Park 2004; Rhymer and Simberloff 1996).

In *Leontopithecus*, hybridization events between *L. chrysomelas* and *L. rosalia*, originating viable and fertile offspring, have already been reported in *ex situ* conditions (Coimbra‐Filho and Mittermeier 1976). Therefore, the illegal introduction of these lion tamarins is very worrying particularly if hybridization occurs. Although hybrids may exhibit intermediate phenotypes to those of their parental species (Smith 1992), a greater representation of genes from a single sire or dam can generate individuals morphologically very similar to just one parental species (Leary et al. 2011), giving rise to cryptic hybrids, as reported for the genus *Callithrix* (Malukiewicz et al. 2021). Thus, complementary analyzes to morphological ones must be implemented to verify the existence of hybrids in areas where a related‐alien species was introduced.

Regardless of hybridization events, an introduced species can pose a significant threat to local species by expanding into areas designated for their conservation (Ruiz‐Miranda et al. 2006). This invasion can compromise the genetic integrity of historically isolated lineages or species (Rhymer and Simberloff 1996). Additionally, non‐native species may adapt to environments where they were not previously suited, sometimes competing with native species (Allendorf et al. 2013). The eventual success of an alien species is possibly due to its potential to be a better competitor than native ones or to the lack of selective pressure, such as the absence of specific pathogens or resistance to local pathogens, in which non‐native species may demonstrate better performance than the native species (Allendorf et al. 2013; Weis 2010).

On the other hand, an introduced species generally suffers an extreme founder effect, resulting from random sampling of alleles from a few individuals belonging to a certain population to establish a new one (Nei et al. 1975). This leads to genetic diversity that is a subset of that original population (Allendorf 1986), furthering rapid genetic erosion (Allendorf and Lundquist 2003; Pérez et al. 2006). In addition, the founder effect may rapidly change allele frequency from one generation to another, causing an increase in the frequency of certain deleterious alleles (Allendorf et al. 2013). Then, genetic drift, which can overcome selection in small populations, may facilitate the fixation of these alleles (Kratzer et al. 2020). Furthermore, if established in a new area, with no subsequent new introductions, the alien species tend to lose fitness by inbreeding (Kratzer et al. 2020), and since the populations usually have a small effective population size, genetic drift will be more crucial and loss of genetic diversity will increase (Brambilla et al. 2015). Therefore, an introduced species may not only pose a threat to native species, but also to its own long‐term survival in that location. Therefore, effective surveillance policies to prevent the illegal introduction of species and also rescue alien species must be part of the scope of governmental actions and strategies to ensure the protection of threatened species.

In this study, we analyzed an allochthonous population of lion tamarins, originating from illegal introductions of *L. rosalia* and *L. chrysomelas* individuals in a protected area of the Brazilian Atlantic Forest, close to the remaining natural distribution range of *L. rosalia*. Our main goal was to investigate the existence of hybrids in order to contribute to the conservation programs and management actions of both species, as well as to infer possible implications related to the founder effect and/or eventual hybridization.

## 2 Material and Methods

### 2.1 Legal and Ethical Requirements

Biological collections followed the ethical and legal standards established by the Ethics Committee in Animal Use and Experimentation of the Federal University of São Carlos (CEUA‐UFSCar, authorization number 7058110316), the Biodiversity Authorization and Information System (SISBIO, MMA, authorization numbers 30939 and 53201), and the Code of best practices for field Primatology (Riley et al. 2014) recommended by the International Primatological Society (IPS) and the American Society of Primatologists (ASP). The access to genetic patrimony was registered at the National Genetic Heritage Management System (SisGen, number A411359).

### 2.2 Background of the Studied Specimens and Area

#### 2.2.1 Serra da Tiririca State Park, Niteroi (RJ, Brazil)

According to the Pri‐Matas Institute, in 1994 there was an illegal release of two males and two females of *L. chrysomelas*, along with one male and one female of *L. rosalia*, in the Serra da Tiririca State Park, a fragment of the Atlantic Forest surrounded by urban areas in the municipality of Niteroi, Rio de Janeiro state (23°70'40.55“E; 74°65'35.3“S), and within the natural distribution range of *L. rosalia* (Figure 1). However, in 2009 a census estimated that approximately 200 non‐native lion tamarins were present in the Niteroi region. All individuals exhibited the phenotype of *L. chrysomelas* (Figure 2) and were confirmed to be fertile after veterinary examinations (A. Pissinati, personal communication).

**Figure 1:**
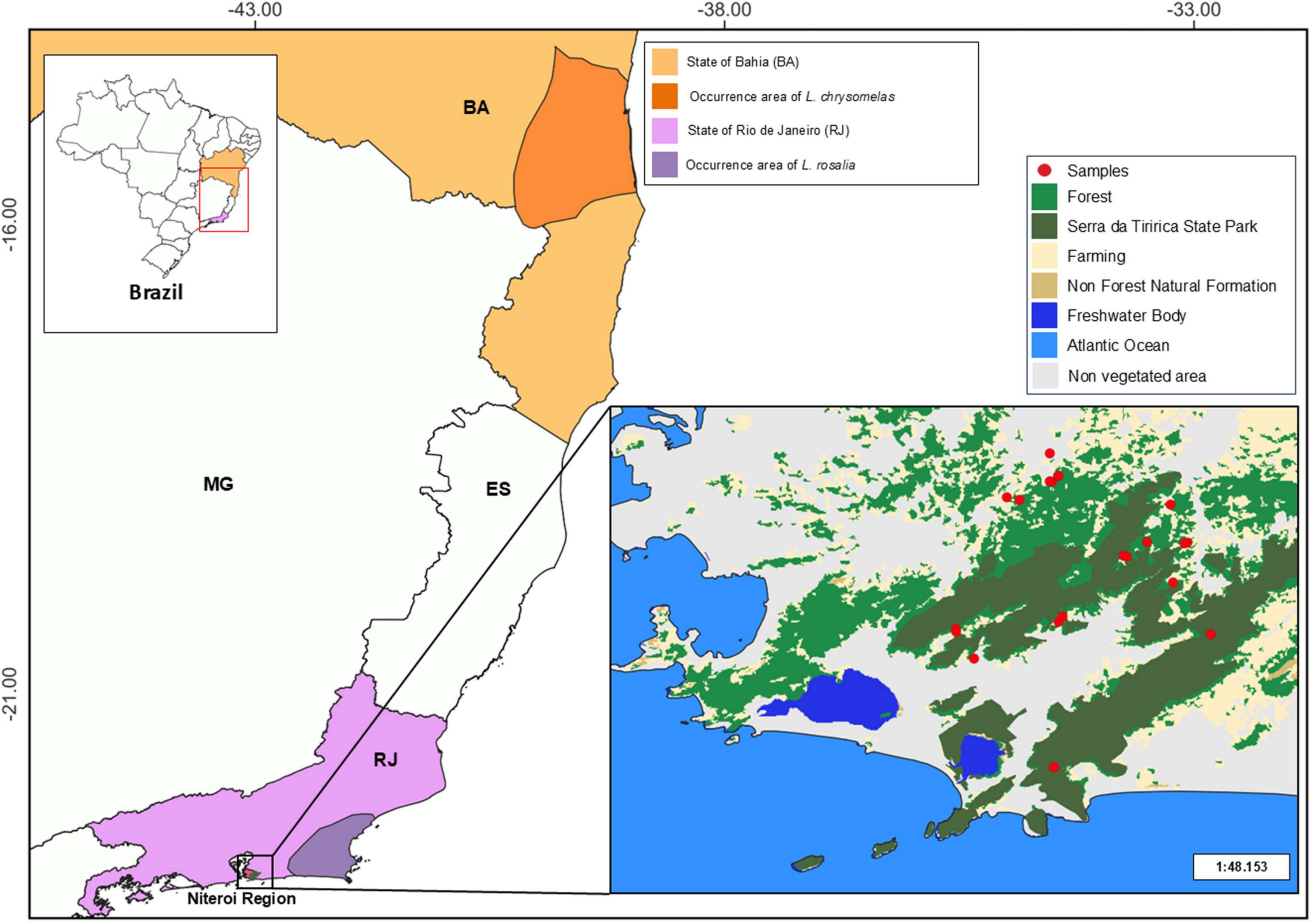
Map illustrating the current distribution of native populations of *Leontopithecus chrysomelas* (GHLT) and *Leontopithecus rosalia* (GLT) as well as the sampling sites of allochthonous lion tamarins in the Serra da Tiririca State Park (Niteroi, Rio de Janeiro), from where the animals studied herein were rescued.

**Figure 2:**
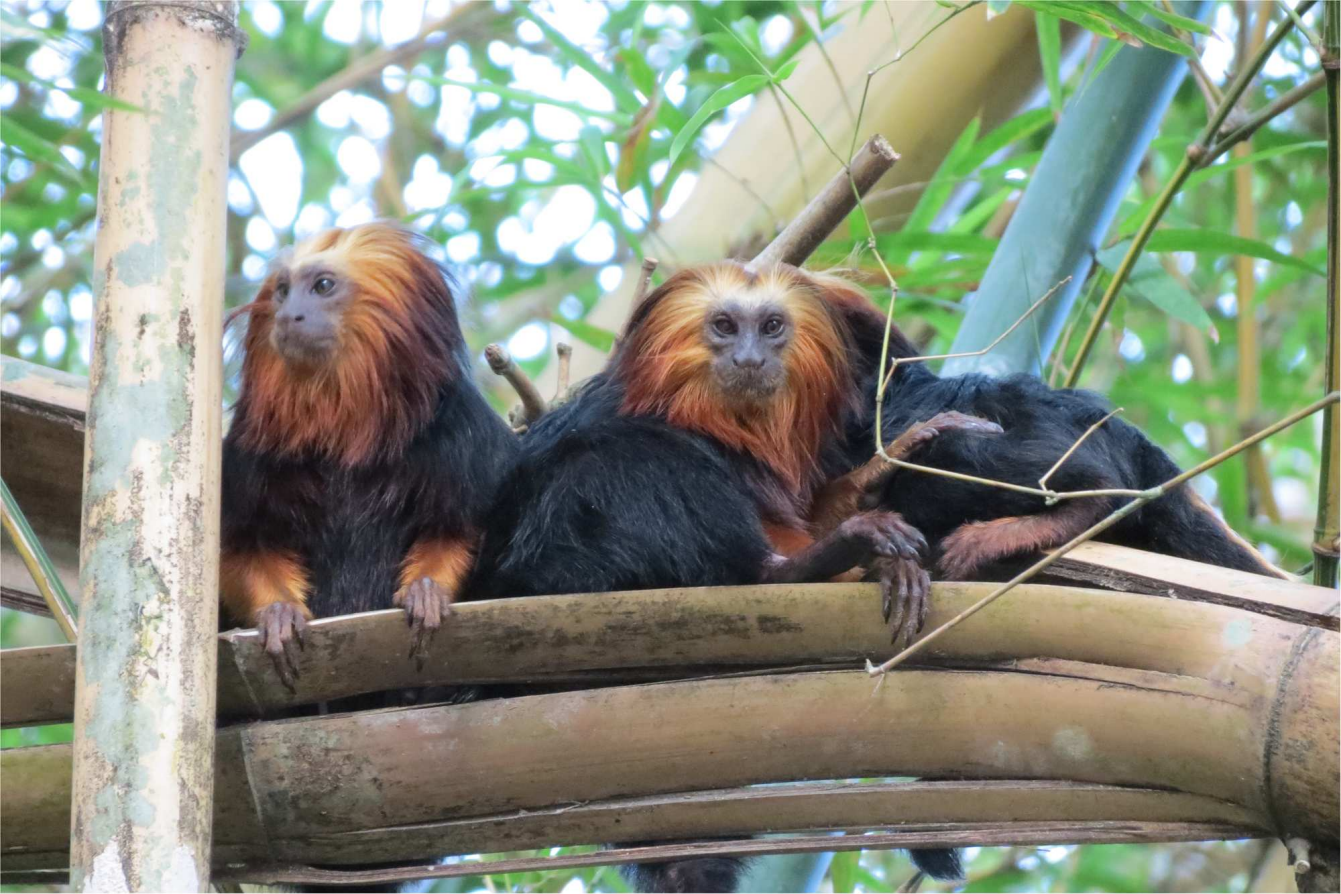
Allochthonous specimens of lion tamarins found in the Serra da Tiririca State Park (Niteroi, Rio de Janeiro, Brazil). Photographed by Danilo Gomes de Freitas Silva.

In the following years, this non‐native population experienced a significant growth. As a result, the Pri‐Matas Institute launched a coordinated conservation effort to relocate and sterilize these lion tamarins, aiming to prevent their expansion into forest fragments where *L. rosalia* currently occurs. From March 2011 to December 2013 approximately 300 alien individuals were translocated to a forest fragment in Bahia state within the original distribution of *L. chrysomelas*, where there were no native individuals. To not overcrowd the forest patch and with no other “empty” forest available, other allochthonous individuals from Niteroi were taken into captivity. Consequently, many individuals from this invasive population were transferred to the Primatology Center of Rio de Janeiro (CPRJ, Guapimirim, RJ), where they were placed under human care in *ex situ* conditions (Kierulff 2015).

### 2.3 Biological Sampling and DNA Extraction

We sampled a total of 44 lion tamarins, comprising seven *L. rosalia*, five *L. chrysomelas*, 31 allochthonous specimens from the Niteroi region, and one captive hybrid individual kept under human care at CPRJ. This hybrid originated under *ex situ* conditions following a natural mating between a *L. chrysomelas* female and a *L. rosalia* male housed in the same enclosure (A. Pissinatti, personal communication). Biological samples of the allochthonous specimens were collected in nine geographic sites surrounding and within the Serra da Tiririca State Park in the Niteroi municipality region (Figure 1). Biological samples of the captive hybrid and the two parental species were acquired from the CPRJ. More detailed information about the biological samples used in this study is provided in the Supporting Material (Table S1).

The procedures for biological sampling of blood and tissue samples of the specimens were carried out by a veterinarian, following the recommendations proposed by the ARRIVE Guidelines (https://arriveguidelines.org/). Briefly, animals were chemically restrained with a combination of ketamin 10% (10 mg/kg ‐ Cetamin® Syntec lab) and midazolam 5 mg/mL (1 mL/kg ‐ Midazolam® ‐ Hipolabor lab) injected intramuscularly. Next, about 0.2 to 0.5 mL of peripheral blood per individual was collected by venipuncture using a *Vacutainer*. During the procedures for biological sampling, the vital signs of the individuals were monitored. In addition, tissue samples of allochthonous specimens were collected during necropsies of individuals that eventually died. For this, tweezers and scissors were used and approximately 200 mg of tissue were collected. Blood and tissue samples were stored, respectively, in EDTA (3.6 mg) and ethyl ethanol (96%), and then kept at −20°C. DNA was extracted from tissue and blood using phenol/chloroform (Wood 1983) and saline solution (Aljanabi 1997), respectively. DNA integrity and quantity were evaluated on a Qubit fluorometer (Life Technologies, Carlsbad, California, United States) and a Nanodrop spectrophotometer (NanoVue Plus, GE Healthcare, Chicago, United States).

### 2.4 Genotyping by Sequencing (GBS) Analyzes

#### 2.4.1 Genomic Library and Sequencing

Genomic libraries were constructed following Elshire et al. (2011). Briefly, 200 ng of each DNA sample were normalized to a concentration of 5 ng/μL using a Qubit fluorometer (Life Technologies, Carlsbad, California, United States) and then digested with the enzyme *Pst*l (New England Biolabs, Ipswich, Massachusetts, United States). Subsequently, ligation reactions were carried out to bind adapters to each DNA sample using T4 DNA ligase. Afterwards, a pool of the samples was purified with magnetic beads (Agencourt AMPure XP‐Beckman Coulter) and amplified *via* Polymerase Chain Reaction (PCR). After restriction and ligation reactions, libraries were selected for fragment sizes between 200 and 450 bp. The library obtained was quantified in a real‐time PCR using the KAPA Biosystems Quantification Kit (Illumina, San Diego, CA, USA). The quality of the libraries was evaluated on an Agilent 2100 BioAnalyzer (Agilent Technology, Santa Clara, CA, USA) using the High Sensitivity DNA Kit (Thermo Fisher Scientific, Waltham, Massachusetts, USA). Final quantification of the library was performed by another real‐time PCR using the KAPA Biosystems Quantification Kit (Illumina, San Diego, CA, USA) and samples were diluted to 2pM. Finally, sequencing was performed, using an Illumina HiSeq. 2500 Platform (Illumina, San Diego, CA, USA).

#### 2.4.2 Processing GBS Data

We performed demultiplexing, quality control, clustering, and variant calling analyses for identification of Single Nucleotide Polymorphisms (SNPs) using the API (iPyRAD analysis tools) available in the pipeline iPyRAD v.0.9.102 (Eaton and Overcast 2020). Briefly, demultiplexing of single‐end reads was performed to individualize the samples, according to the combination of barcode indexes used. After the individualization of each sample, adapter, and barcode sequences were trimmed, and reads were filtered for Phred quality QScore < 20. Only reads larger than 35 bp were retained. Then, sequences were aligned with the reference genome of *L. chrysopygus* (unpublished data) and pooled using a 90% sequence similarity threshold within and between samples to identify SNPs and orthologous loci, with a minimum depth of 6 reads per locus. The minimum number of samples per locus was set at 70%. A final data filtering step was conducted using the R package *r2vcftools* (Danecek et al. 2011). First, only bi‐allelic loci were maintained, then, those individuals with more than 70% of missing data were excluded. All loci were tested for excess heterozygosity, as indicated by significant deviations from Hardy‐Weinberg Equilibrium (HWE, *p* < 0.0001). Filtering criteria also included a minimum minor allele frequency (MAF) of 0.05%, linkage disequilibrium (LD, r2 < 0.8), and the presence of loci in at least 70% of the samples. It is important to emphasize that all Niteroi samples were obtained from tissue samples, which minimizes the potential impact of eventual chimerism on genetic diversity estimates (Takabayashi and Katoh 2015).

#### 2.4.3 Detection of Putative Neutral and Outliers SNPs

For the identification of putative neutral and outlier loci, we performed a Principal Component Analysis (PCA), based on Mahalanobis distance with PCAdapt v.4.3.3 (Luu et al. 2017) in R (R Core Team 2020). Outliers were determined according to false discovery rate (FDR) of 0.05, with the R package q‐value (Storey and Tibshirani 2003), with K set to 3. Neutral SNPs were subsequently used to assess genetic diversity, infer population structure, and then to search for signs of hybridization.

#### 2.4.4 Population Structure and Genetic Diversity

We used the Adegenet package (Jombart 2008) in R (R Core Team 2020), to perform a multivariate approach based on the Discriminant Analysis of Principal Components (DAPC) and implement a Principal Component Analysis (PCA). Sampling areas were not considered as priors. We used alpha score optimization to determine the best number of PCs to retain by considering PCs that accounted for 80% of the variance. To determine the best number of discriminant components, the Bayesian Information Criterion (BIC) was used. Next, we employed a non‐negative matrix factorization (sNMF) and the least squares optimization procedure of the ancestry coefficients to estimate the individual ancestry and population clusters using the function *snmf* in the R package LEA (Frichot and François 2015). This function was used with 100 repetitions for each value of k, ranging from 1 to 10. To determine the best k value to explain the results, the cross‐entropy criterion was adopted.

The pairwise F_ST_ was calculated using the *genet.dist* function. To obtain the confidence interval, we used the *pwfst* function of the R package with 1,000 bootstrap replications (Gruber et al. 2018). The effective population size (N_E_) for species and genetic subgroups was estimated using the Linkage Disequilibrium (LD) approach with a random mating system, and considering MAF = 0.10, as implemented in NeEstimator v.2 (Do et al. 2014), using dartR (Gruber et al. 2018).

We also used *F*_3_‐statistics to formally test for admixture using the 3PopTest from AdmixTools (Patterson et al. 2012; Peter 2016). This test detects if population C is admixed between source populations A and B. If *f*_3_(C; A, B) is significantly negative, this suggests that broadly the allele frequency of population C is intermediate to the frequencies of A and B, and thus it is likely to represent an admixed population. If the statistic is positive, this suggests that population C is unadmixed. It is worth noting that strong population‐level drift post‐admixture can mask the signal and could result in a non‐negative statistic even when admixture occurred (Patterson et al. 2012). Statistical significance is assessed by a Z‐score of magnitude three or higher as calculated by a block jackknife approach.

To estimate genetic diversity parameters, we used the Hierfstat package (Goudet 2005), considering the genetic groups assigned by the PCA and sNMF analyses. We calculated the observed (H_O_) and expected (H_E_) heterozygosity, and F_IS_ values, using the *basic. stats* function. To calculate the intervals of confidence for the population‐specific F_IS_ we used the *boot. ppfis* function, with 1,000 bootstrap replicates. To compare expected heterozygosity (H_E_) between populations with unequal sample sizes, we applied a resampling approach to standardize sample sizes. For each pairwise comparison, the population with the larger sample size was randomly subsampled to match the size of the smaller population. This process was repeated for 1,000 iterations, retaining only loci with at least two genotyped individuals. Expected heterozygosity was then estimated using the *basic. stats* function from the Hierfstat R package. The mean H_E_ of the 1,000 subsamples from the Niteroi population, considering *N* = 3 and *N* = 7, was then compared to the H_E_ obtained for the analyzed populations considering the total sample sizes. To assess statistical significance, we calculated the *p*‐value as the proportion of subsamples with H_E_ equals to or greater than that of the smaller population sizes.

### 2.5 Mitochondrial Analysis

Polymerase chain reactions (PCRs) were performed for the amplification of the Cytochrome Oxidase I subunit I (COI) gene, using the primer pair described by Folmer et al. (1994). PCRs were carried out using 1.0 U of Taq, 2.5 mM MgCl2, 50 mM KCl, 10 mM Tris‐HCl, 0.2 mM dNTPs and 50 ng of DNA in a final volume of 10 µL. The amplification reactions were performed on an Eppendorf Mastercycler Gradient® thermal‐cycler (Eppendorf AG, Hamburg, Germany) under the specific conditions: an initial denaturation cycle at 95°C for 6 min, followed by 35 cycles of 94°C for 1 min, 60°C 45 s and 72°C for 45 s, and a final extension step at 72°C for 10 min.

PCR products were analyzed on agarose gel 2% with GelRed, under a UV light transilluminator (Uniscience ETX35M, Vilmer Lourmat, Collégien, France). The amplified fragments were then purified following the protocol proposed by Lis (1980), and then sequenced on an equipment ABI 3730XL (Applied Biosystems, Waltham, Massachusetts, USA). The obtained sequences for one captive hybrid, four *L. rosalia*, four *L. chrysomelas* and 11 allochthonous individuals randomly sampled from the Niteroi population (Table S1) were aligned using the ClustalW tool, available in Geneious (Kearse et al. 2012).

A network haplotype was constructed using PopArt (Leigh and Bryant 2015), with the Median‐Joining method (Bandelt et al. 1999). A Bayesian inference tree was constructed in BEAST v.2.7.7 (Bouckaert et al. 2019) using the HKY inferred with jModelTest v.2.1.10 (Darriba et al. 2012) and the Bayesian Information Criterion (BIC). Three independent runs with 50,000,000 generations (Markov Chain Monte Carlo, MCMC), sampling every 5,000,000 using a strict clock, and the Yule Model. The convergence of the chains and the effective sampling size (ESS) values for the estimated parameters were checked in Tracer v.1.7.1 (Rambaut et al. 2018), considering effective sampling size (ESS) values of 200 or more for all parameters. A consensus tree was combined and resampled in the LogCombiner v.1.8.2 software, considering 10% burn‐in and then summarized in TreeAnnotator and visualized in FigTree v.1.4.4. A COI sequence for a specimen of *Callithrix jacchus* (Access Number: JF444285.1) was downloaded from GenBank (https://www.ncbi.nlm.nih.gov/genbank/) and used as an outgroup.

## 3 Results

### 3.1 SNPs Data

The genomic library sequencing resulted in a total of 122,524,900 raw reads for 44 lion tamarins. After raw data processing using iPyRAD, we obtained an initial catalogue of 36,705 SNPs for a total of 42 individuals, from which 36,579 bi‐allelic loci were selected. Then, samples with more than 70% of missing data (408, 421, 466, 471) were excluded. After final filtering, we recovered a total of 5,959 bi‐allelic loci for 38 individuals, including 27 specimens from Niteroi, seven *L. rosalia*, three *L. chrysomelas* and the captive hybrid. From this catalogue, 1,889 loci were outliers, and 4,070 loci were considered neutral and were used in the subsequent population analyses.

The sNMF analysis using only neutral SNPs evidenced two ancestral genetic groups (*K* = 2), based on the lowest cross‐entropy criterion, with the captive hybrid (H) presenting approximately 50% of membership from each *L. rosalia* (GLT) and *L. chrysomelas* (GHLT) species. All individuals of *L. rosalia* showed the same probability of belonging to a single genetic cluster, while all individuals of *L. chrysomelas* showed similar membership proportions (Figure 3A,B). Principal Component Analysis (PCA) also evidenced specimens of *L. rosalia* clustered together in a different axis of the specimens from Niteroi, which were clustered together in the same component along with the captive specimens of *L. chrysomelas*. On the other hand, the captive hybrid appeared allocated in a more central position between the two PCs (Figure 3C). The F_ST_ values corroborate these results, showing a very low value for the fixation index (F_ST_ = 0.158) between Niteroi specimens and *L. chrysomelas*. Conversely, *L. rosalia* evidenced higher fixation values when compared to *L. chrysomelas* from captivity (F_ST_ = 0.547) and Niteroi (F_ST_ = 0.443) (Figure 3D). When captive *L. chrysomelas* and individuals from Niteroi were grouped and compared to *L. rosalia*, a high fixation index (F_ST_ = 0.419) was also obtained. The population analysis using 1,889 outlier SNPs also showed evidence of similar structure results, but suggested a sub‐structuring within *L. rosalia*, possibly due to the distinct family groups kept in captivity (Figure S1).

**Figure 3:**
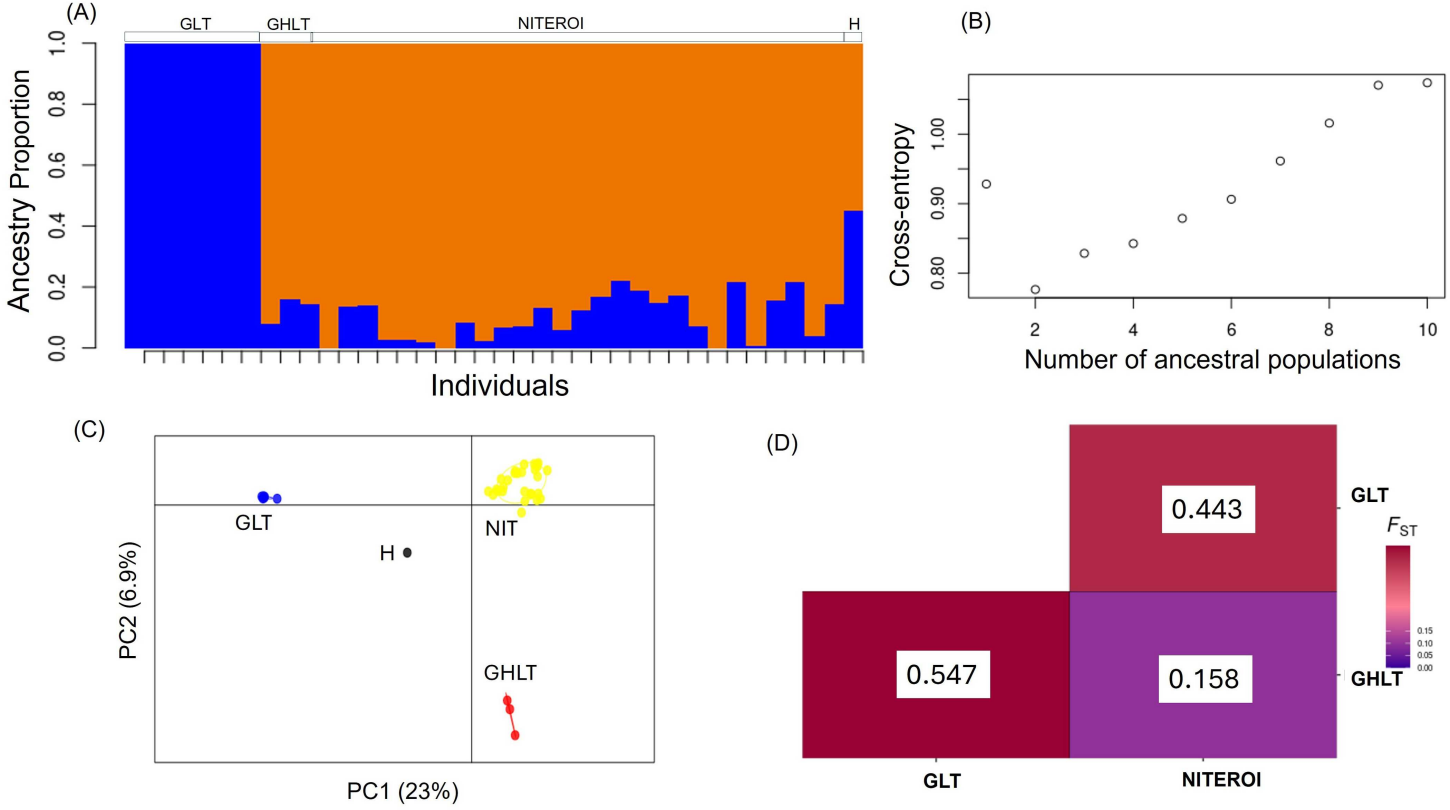
Genetic population structure using 4,070 neutral SNPs obtained for *Leontopithecus chrysomelas* (GHLT), *Leontopithecus rosalia* (GLT), individuals from the allochthonous population of Niteroi (NIT), and a captive hybrid (H), based on (A) a non‐negative matrix factorization (sNMF) approach and (B) its cross‐entropy (*K* = 2); (C) principal component analysis (PCA); and (D) fixation index (F_ST_) estimates.

For the known hybrid compared to the source samples of *L. rosalia* and *L. chrysomelas*, we found a significantly negative *F*_3_‐statistic (Table 1). This confirms our ability to use this method to identify admixture in this dataset and aligns with our expectations regarding the nature of the hybridization event between the two species in captivity. For the individuals from Niteroi compared to the same source samples, we found a significantly positive *F*_3_‐statistic value (Table 1). This result indicates that the allochthonous population did not arise from admixture between the two species, corroborating the previous analyses.

**Table 1:** Significant (|*Z* | > 3) *F*_3_‐statistics for Niteroi population (*N* = 25) and known hybrid (*N* = 1) compared to source samples of *L. rosalia* (*N* = 7) and *L. chrysomelas* (*N* = 3). SE = standard error.

Target	Source 1	Source 2	*f*_3_	SE	Z	SNPs
Niteroi	*L. rosalia*	*L. chrysomelas*	0.088138	0.007423	11.874	2305
Hybrid	*L. rosalia*	*L. chrysomelas*	−0.225779	0.006971	−32.391	1543

*Note:* *Removed 2 additional samples with more than 40% missingness.

Concerning the genetic diversity parameters, comparable values of allelic richness, ranging from 1.12 to 1.38, were found for all species and/or populations analyzed herein (Table 2). However, the expected heterozygosity for *L. rosalia* (H_E_ = 0.124) was smaller when compared to that found for *L. chrysomelas* from *ex situ* conditions (H_E_ = 0.369) and from the Niteroi population (H_E_ = 0.376). The Niteroi population and the samples of *L. chrysomelas* from CPRJ showed similar expected heterozygosity estimates. A slightly significant heterozygosity excess was found for *L. chrysomelas* from captivity, which evidenced higher values of observed than expected heterozygosity. On the other hand, the Niteroi population exhibited observed heterozygosity lower than expected (Table 2). We were not able to estimate the effective population size (N_E_) for *L. rosalia* and *L. chrysomelas* from CPRJ due to the small number of individuals sampled, however, the Niteroi population showed a relatively high estimate (N_E_ = 26.2, 95% CI: 26.0−26.4).

**Table 2:** Genetic diversity estimates using 4,070 neutral SNPs obtained for *Leontopithecus chrysomelas* (GHLT), *Leontopithecus rosalia* (GLT), and allochthonous lion tamarins from Niteroi. N: number of sampled individuals; A_R_: allelic richness; H_O_: observed heterozygosity; H_E_: expected heterozygosity; F_IS_: inbreeding coefficient; and F_IS_CI: confidence interval.

Samples	*N*	A_R_	H_O_	H_E_	F_IS_	F_IS_CI
*Leontopithecus rosalia*	7	1.12	0.121	0.124	0.009	−0.002−0.053
*Leontopithecus chrysomelas*	3	1.37	0.374	0.369	−0.063^a^	−0.043−0.003
Lion tamarins of Niteroi	27	1.38	0.347	0.376	0.084^a^	0.088−0.106

^a^ Statistically significant values.

When we considered subsamples (N_S_) with standardized population sizes, the mean expected heterozygosity of the Niteroi population (H_E_ = 0.378, N_S_ = 3) was similar (*p* = 0.548) to that found for *L. chrysomelas* from CPRJ (H_E_ = 0.369, *N* = 3). Conversely, the Niteroi population showed a higher (*p* = 0.001) mean expected heterozygosity (H_E_ = 0.377, N_S_ = 7) when compared to *L. rosalia* (H_E_ = 0.124, *N* = 7). Also, *L. rosalia* showed a smaller (*p* = 0.001) value of mean expected heterozygosity (H_E_ = 0.011, N_S_ = 3) when compared to *L. chrysomelas* from CPRJ (H_E_ = 0.369, *N* = 3).

### 3.2 Mitochondrial Data

COI gene amplifications produced sequences ranging from 684 to 703 bp. After alignment and editing, we obtained fragments of 571 bp, evidencing 15 polymorphic sites among *L. chrysomelas* and *L. rosali*a. The results distinguished both species and confirmed the maternal contribution of *L. chrysomelas* to the captive hybrid. The phylogenetic tree evidenced the specimens from Niteroi and *L. chrysomelas* clustered together, while the haplotype network showed common haplotypes among them (Figures 4, 5 and S2).

**Figure 4:**
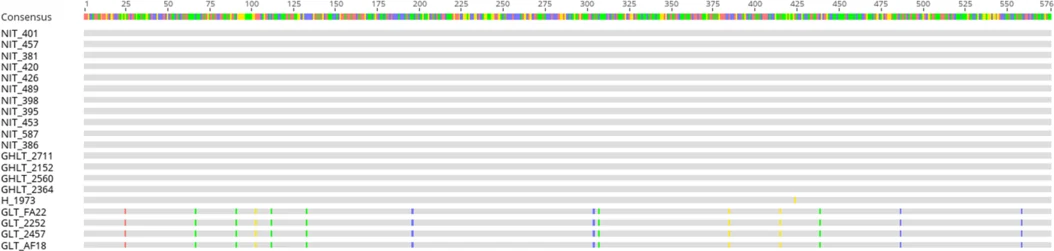
Alignment using COI data, showing the polymorphic sites among samples of allochthonous lion tamarins found in Niteroi (Niteroi), *Leontopithecus chrysomelas* (GHLT), *Leontopithecus rosalia* (GLT), and a captive hybrid (H) between the two parental species.

**Figure 5:**
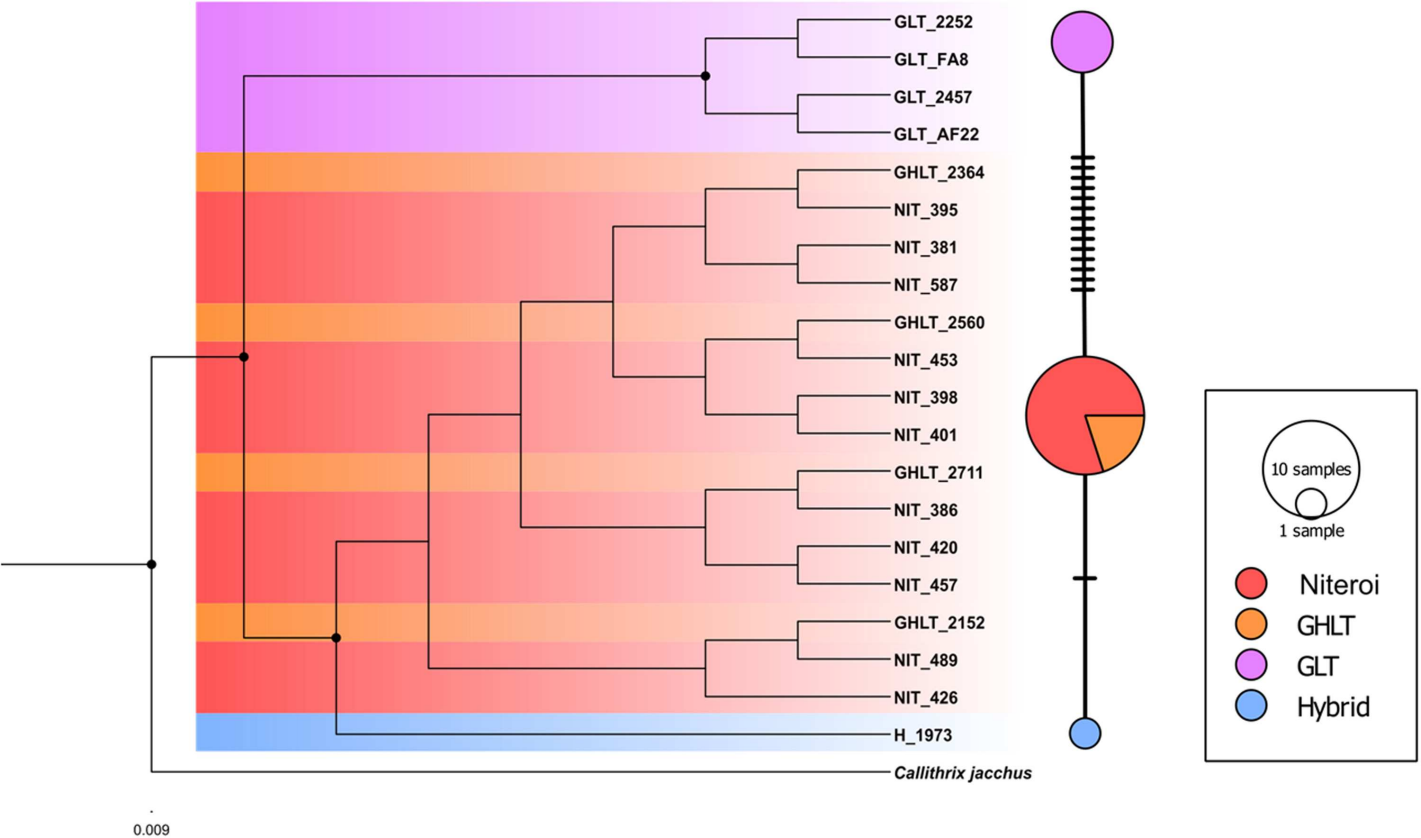
Bayesian phylogenetic tree (A) and haplotype network (B) using COI data, showing, respectively, the genetic relationship and clades, and the three haplogroups found for the samples of allochthonous lion tamarins from Niteroi (NIT), *Leontopithecus chrysomelas* (GHLT), *Leontopithecus rosalia* (GLT), and a captive hybrid between the two species (H). The species *Callithrix jacchus* (JF444285.1) was used as an outgroup in the phylogenetic tree. Nodes with a black circle indicate Bayesian posterior probability above 0.95.

## 4 Discussion

Based on the genomic approach and complementary mitochondrial analyses employed in this study, we found no evidence of recent hybridization in the lion tamarins from the Niteroi region, an area allochthonous to the geographic distribution range of *L. chrysomelas* and within the natural occurrence of *L. rosalia* (Oliveira et al. 2021; Ruiz‐Miranda et al. 2021). Mitochondrial analyses clearly distinguish both species, and the SNP and COI datasets showed no evidence of *L. rosalia* in the area, indicating that the introduced individuals are pure *L. chrysomelas*. In addition, the genomic analysis estimated a relatively higher genetic diversity for the invasive population, raising concerns about its persistence in an area close to the current occurrence of *L. rosalia*.

The population analyses using SNPs showed that captive *L. chrysomelas* and the specimens from Niteroi have similar ancestry proportions to each other, and they are different from *L. rosalia* and from the captive hybrid, which presented an approximate proportion of fifty percent of the genome of each of the parental species. The mitochondrial analysis confirmed the maternal contribution to the captive hybrid from *L. chrysomelas*. The fixation index indicated a very slight differentiation among the specimens of Niteroi and *L. chrysomelas* kept in *ex situ* condition. At the level of species, such a low value (F_ST_ = 0.158) should be considered a low‐moderate population substructuring (Wright 1978). Conversely, the fixation index between *L. rosalia* and Niteroi population specimens was quite high (F_ST_ = 0.443), suggesting a high genetic differentiation among them, and reinforcing also the sNMF and the PCA results that showed no signs of hybridization between *L. chrysomelas* and *L. rosalia*.

Previous studies using enzymes, nuclear and/or mitochondrial markers have pointed out *L. rosalia* and *L. chrysomelas* as very closely related species (Moreira et al. 1996; Mundy and Kelly 2001; Perez‐Sweeney et al. 2008). Although there are few approaches analyzing the four species of the genus, genetic relationships between some of them have been reported *L. chrysomelas* as the basal taxon within the genus (Buckner et al. 2015; Perez‐Sweeney et al. 2008). Thus, the small proportion of genome shared between *L. rosalia* and *L. chrysomelas* may be reflection of this phylogenetic relationship, justifying our findings for both species, including the specimens from Niteroi, which are here assumed to be *L. chrysomelas*. Indeed, the F3 statistics corroborated the absence of hybrids among the allochthonous specimens of Niteroi. Therefore, we emphasize that no signs of hybridization were detected in the allochthonous lion tamarins of Niteroi.

Although our findings do not raise concerns about how fertile hybrids may negatively impact their parental species, the allochthonous specimens of Niteroi continue to pose a threat to *L. rosalia*. This alien population has been shown to have adapted very well to the area, reproducing regularly, and increasing its population enormously, reaching more than a thousand animals in the last two decades (C. Kierulff, personal communication). Due to this massive growth in population size, and the risk that the expansion of *L. chrysomelas* represents for the native *L. rosalia*, conservation management actions have been carried out to translocate the alien lion tamarins. Thus, many lion tamarins of Niteroi were transferred to the CPRJ, and approximately 300 allochthonous individuals were transferred to a forest fragment in a Private Reserve owned by Veracel Celulose (24°45'76.67“E; 82°30'65.0“S), located in Belmonte, Bahia state, within the natural distribution range of *L. chrysomelas* (Kierulff 2015).

The legally coordinated conservation effort to transfer the lion tamarins from Niteroi integrated multiple efforts, bringing together Brazilian and international agencies to rescue the animals in different locations in the metropolitan region, carry out sanitary procedures and then transport them by plane to finally release them safely into the wild (Kierulff 2015). Despite this huge effort, invasive *L. chrysomelas* individuals can still be found in the Niteroi region to date and the effort to remove them is still ongoing (C. Kierulff, personal communication).

The ability of these individuals to easily adapt, reproduce, and then expand in population size may allow them to overcome geographic isolation, potentially increasing their range, which could facilitate future competition and/or hybridization with the closest population of *L. rosalia*, located less than 50 km away (Kierulff 2015). This is evidenced by the relatively high value of effective size (*N*_E_ = 26), estimated for a population founded by only two couples. Population expansion of allochthonous species has already been reported for other callitrichids, such as *C. jacchus* and *Callithrix penicillata*, which were introduced into the Atlantic Forest in Rio de Janeiro state, threatening local species and promoting artificial hybridization zones (Rocha et al. 2004).

Regarding the absence of *L. rosalia* among the analyzed animals, it is difficult to infer what happened in terms of the viability and reproduction of the introduced couple of this species in the 1990's. However, if *L. rosalia* produced offspring, they are no longer in the Niteroi region. No specimens with the *L. rosalia* phenotype were captured or observed during the rescue of lion tamarins in Niteroi, which began in 2011 (Kierulff 2015) and was extended in subsequent years to monitor this invasive population to date. Also, our results did not show any genomic evidence to assign *L. rosalia* among the allochthonous specimens analyzed herein. This supports the suggestion that *L. chrysomelas* may have replaced *L. rosalia* during the demographic expansion of the invasive population in that region.

Species replacements have been described as a phenomenon commonly observed when native and non‐native species compete for resources and habitat (Allendorf et al. 2013). Alien species may succeed native ones due to the novel environmental conditions or specific traits inherent to these species, which facilitate their population growth in the new habitat (Parker et al. 2013), as reported for the Neotropical primate *Callithrix aurita* (Carvalho et al. 2019). This endangered species is also endemic to the Brazilian Atlantic Forest and has been suffering negative impacts due to the presence of invasive *Callithrix* specimens and their hybrids, which are outcompeting and replacing native populations throughout its geographic distribution range (Carvalho et al. 2019).

Considering the scenarios of absence of *L. rosalia* and hybrids in Niteroi region and based on historical records and field observation that report no new introduction of lion tamarins in the area (C. Kierulff, personal communication), the foundation of this invasive population would come from only two males and two females of *L. chrysomelas*. However, the expected heterozygosity found for the invasive population using the total sampling was relatively high (0.376). This value was significantly higher than that of *L. rosalia* (0.124) and similar to that found for *L. chrysomelas* (0.369) kept in *ex situ* conditions. On the other hand, the values of allelic richness among them are all comparable, even when using different sampling sizes. When we performed random subsampling for the allochthonous specimens, the mean expected heterozygosity, considering population sizes equal to those sampled for *L. rosalia* (*N* = 7) and captive individuals of *L. chrysomelas* (*N* = 3) from CPRJ, was comparable to that using the total sample size of the Niteroi population (*N* = 27).

Since the present study is the first one using SNPs to characterize genetic diversity of both *ex situ* and *in situ* populations of *L. chrysomelas*, and also of *L. rosalia*, there is no SNP information on other *Leontopithecus* species for any comparison. However, very low levels of SNP‐based heterozygosity (0.034−0.069) have been reported for the endangered snub‐nosed monkeys *Rhinopithecus* (Kuang et al. 2020). Indeed, population genomic studies are scarce for Neotropical primates to date and genomic data are often restricted to approaches that address mainly evolutionary questions using sampling generally limited to one individual per species (Hermosilla‐Albala et al. 2024, 2025; Kuderna et al. 2023; Shao et al. 2023).

Inferences based on SNPs derived from drafts of whole genomes have shown very low levels of autosomal heterozygosity for primates from the family Pitheciidae, such as those from the genus *Cacajao* (0.0014−0.0025) (Hermosilla‐Albala et al. 2024), and from the family Atelidae, such as *Brachyteles hypoxanthus* (0.0044) and *Ateles paniscus* (0.0013) (Hermosilla‐Albala et al. 2025). For the Callitrichidae family, values of autosomal heterozygosity lower than 0.002 have been reported through whole genome analyses (Kuderna et al. 2023). Therefore, our findings contribute to increasing knowledge about genomic diversity assessed from large‐scale SNP datasets in threatened Neotropical primates and suggest that the invasive population of Niteroi has maintained or recovered its heterozygosity over generations.

Natural genetic diversity recovery has been observed in some threatened species especially after suffering bottleneck events, such as the Tibetan antelope (Du et al. 2016) and the Scandinavian arctic fox (Nyström et al. 2006). Strong bottlenecks usually result in the loss of genetic diversity, however, rapid growth of a small or founder population can mitigate genetic erosion and foster adaptive changes (Allendorf et al. 2013; Blanchong et al. 2013).

Populations that experienced a recent bottleneck typically display an excess of heterozygosity, with observed heterozygosity exceeding expected heterozygosity (Luikart and Cornuet 1998), as the *ex situ* population of *L. chrysomelas* analyzed herein. Furthermore, *ex situ* populations may present higher observed heterozygosity than expected due to assisted genetic management to avoid increased inbreeding and consequent loss of fitness (Frankham et al. 2008), as already reported for captive populations of both *L. chrysopygus* and *L. chrysomelas* (Aliaga‐Samanez et al. 2023; Ayala‐Burbano et al. 2017; Javarotti et al. 2025). On the other hand, a heterozygote deficit can be related to an increase in the inbreeding rate commonly observed in small populations that interbreed randomly (Frankham et al. 2008). Thus, the subtle heterozygosity deficit observed in the allochthonous population of *L. chrysomelas* can be the result of a potential inbreeding during the initial population expansion process. Overall, although our findings indicate the likely maintenance of genetic diversity due to the expansion of the allochthonous population in Niteroi, the inbreeding coefficient suggests a tendency for interbreeding between genetically more closely related individuals. It is also important to emphasize that we cannot rule out unknown additional introductions, since distinct events of illegal introductions over the years could also explain the high genetic diversity and the estimated effective population size.

## 5 Conclusions

The present study sheds light on the question of hybridization between *L. rosalia* and *L. chrysomelas* in the Niteroi region, indicating the absence of hybrids among the analyzed samples from the allochthonous population. This research establishes an efficient SNP‐based protocol for identifying hybrids between these endangered species, serving as a model study for further approaches aiming to investigate hybridization within *Leontopithecus*. Furthermore, this is the first study using large‐scale SNP datasets to assess genetic diversity parameters in *ex situ* and *in situ* populations of species from the endangered genus *Leontopithecus*. The data shows that the estimated genetic diversity of *L. chrysomelas* tends to be higher than that of *L. rosalia*, which has implications for the future conservation of *L. rosalia*, given ongoing concerns about species replacement. Our results are relevant to aid further decision‐making on the management of the studied allochthonous population and for the conservation programs of both *L. rosalia* and *L. chrysomelas* species.

## Author Contributions

**Gabriela Aliaga–Samanez:** Writing – review & editing, Writing original draft, Methodology, Formal analysis, Investigation, Data curation, Conceptualization, Visualization. **Carla Gestich:** Methodology, Formal analysis, Writing – review & editing, Data curation. **Paola Andrea Ayala‐Burbano:** Formal analysis, Writing – review & editing. **Nathalia Bulhões Javarotti:** Methodology, Formal analysis, Data curation. **Katherine McVay:** Writing – review & editing, Formal analysis. **Maria Cecilia Martins Kierulff:** Writing – review & editing. **Ana Paula Cazerta Farro:** Writing – review & editing. **Sílvia Bahadian Moreira:** Writing – review & editing. **Alcides Pissinatti:** Writing – review & editing. **Amy Goldberg**: Writing – review & editing; Funding acquisition. **Pedro M. Galetti Jr.**: Writing – review & editing. **Patrícia Domingues de Freitas:** Writing – review & editing, Supervision, Investigation, Funding acquisition, Conceptualization, Visualization. [Correction added on 10 February 2026, after first online publication: The author contributions have been updated to include the complete details, which were previously missing from the original published version.]

## Supporting information


**Figure S1:** Genetic population structure using 1,889 outlier SNPs of Leontopithecus chrysomelas (GHLT), Leontopithecus rosalia (GLT), and a captive hybrid (H) from the Primatology Center of Rio de Janeiro (CPRJ) and individuals from the allochthonous population of Niteroi (NIT), based on (A) a non‐negative matrix factorization (sNMF) approach and (B) its cross‐entropy (K = 2); (C) principal component analysis (PCA); and (D) fixation index (FST) estimates.

**Figure S2:** Alignment for the COI fragments (571 bp) obtained for Leontopithecus chrysomelas (GHLT), Leontopithecus rosalia (GLT), one captive hybrid between these species (H) from the Primatology Center of Rio de Janeiro (CPRJ), and allochthonous specimens of lion tamarins from Niteroi (Rio de Janeiro, Brazil).

Supporting Material ‐ Tables ‐ Review.
