# Supplementary material for: To be or Not to be Hybrids? A Case‐Study in an Allochthonous Population Involving Illegal Introductions of the Endangered Golden‐Headed and Golden Lion Tamarins
Source: Am J Primatol. 2025 Dec 31;88(1):e70112. doi: 10.1002/ajp.70112 (PMC13749594; doi:10.1002/ajp.70112)
Supplement: Supplementary file 1 [file AJP-88-e70112-s001.docx]

**Supplementary Material**

**Table S1.** Data summary for the analyzed individuals of *Leontopithecus sp.* from Niteroi (NIT), *Leontopithecus rosalia* (GLT), *Leontopithecus chrysomelas* (GHLT), and a captive hybrid between GLT and GHLT from the Primatology Center of Rio de Janeiro (CPRJ, RJ). Positive (x) and negative (-) results obtained for genotyping by sequencing (GBS) and Cytochrome Oxidase I (COI) analyses.

| **ID** | **Specie** | **Sex** | **Collection year** | **Origin** | **Sample** | **GBS** | **COI** |
| --- | --- | --- | --- | --- | --- | --- | --- |
| GLT_2252 | *L. rosalia* | Female | 2014 | CPRJ | Blood | X | X |
| GLT_2457 | *L. rosalia* | Male | 2014 | CPRJ | Blood | X | X |
| GLT_FA8 | *L. rosalia* | Male | 2015 | CPRJ | Blood | X | X |
| GLT_AF22 | *L. rosalia* | Female | 2015 | CPRJ | Blood | X | X |
| GLT_3673 | *L. rosalia* | Female | 2020 | CPRJ | Blood | X | - |
| GLT_3794 | *L. rosalia* | Female | 2020 | CPRJ | Blood | X | - |
| GLT_3863 | *L. rosalia* | Female | 2020 | CPRJ | Blood | X | - |
| GHLT_2560 | *L. chrysomelas* | Male | 2015 | CPRJ | Blood | - | X |
| GHLT_2711 | *L. chrysomelas* | Female | 2015 | CPRJ | Blood | - | X |
| GHLT_2152 | *L. chrysomelas* | Female | 2014 | CPRJ | Blood | X | X |
| GHLT_2364 | *L. chrysomelas* | Female | 2014 | CPRJ | Blood | X | X |
| GHLT_1894 | *L. chrysomelas* | Male | 2014 | CPRJ | Blood | X | - |
| H_1973 | Hybrid | Female | 2014 | CPRJ | Blood | X | X |
| NIT_365 | *Leontopithecus sp.* | Female | 2013 | Niteroi | Tissue | X | - |
| NIT_460 | *Leontopithecus sp.* | Male | 2013 | Niteroi | Tissue | X | - |
| NIT_454 | *Leontopithecus sp.* | Female | 2013 | Niteroi | Tissue | X | - |
| NIT_371 | *Leontopithecus sp.* | Female | 2013 | Niteroi | Tissue | X | - |
| NIT_381 | *Leontopithecus sp.* | Male | 2013 | Niteroi | Tissue | X | X |
| NIT_430 | *Leontopithecus sp.* | Female | 2013 | Niteroi | Tissue | X | - |
| NIT_401 | *Leontopithecus sp.* | Female | 2013 | Niteroi | Tissue | X | X |
| NIT_453 | *Leontopithecus sp.* | Female | 2013 | Niteroi | Tissue | X | X |
| NIT_420 | *Leontopithecus sp.* | Male | 2013 | Niteroi | Tissue | X | X |
| NIT_408 | *Leontopithecus sp.* | Female | 2013 | Niteroi | Tissue | X | - |
| NIT_421 | *Leontopithecus sp.* | Male | 2013 | Niteroi | Tissue | X | - |
| NIT_449 | *Leontopithecus sp.* | Female | 2013 | Niteroi | Tissue | X | - |
| NIT_426 | *Leontopithecus sp.* | Female | 2013 | Niteroi | Tissue | X | X |
| NIT_398 | *Leontopithecus sp.* | Female | 2013 | Niteroi | Tissue | X | X |
| NIT_414 | *Leontopithecus sp.* | Male | 2013 | Niteroi | Tissue | X | - |
| NIT_386 | *Leontopithecus sp.* | Male | 2013 | Niteroi | Tissue | X | X |
| NIT_431 | *Leontopithecus sp.* | Male | 2013 | Niteroi | Tissue | X | - |
| NIT_379 | *Leontopithecus sp.* | Male | 2013 | Niteroi | Tissue | X | - |
| NIT_457 | *Leontopithecus sp.* | Male | 2013 | Niteroi | Tissue | X | X |
| NIT_587 | *Leontopithecus sp.* | Male | 2013 | Niteroi | Tissue | X | X |
| NIT_489 | *Leontopithecus sp.* | Male | 2013 | Niteroi | Tissue | X | X |
| NIT_466 | *Leontopithecus sp.* | Female | 2013 | Niteroi | Tissue | X | - |
| NIT_462 | *Leontopithecus sp.* | Female | 2013 | Niteroi | Tissue | X | - |
| NIT_479 | *Leontopithecus sp.* | Male | 2013 | Niteroi | Tissue | X | - |
| NIT_471 | *Leontopithecus sp.* | Male | 2013 | Niteroi | Tissue | X | - |
| NIT_473 | *Leontopithecus sp.* | Male | 2013 | Niteroi | Tissue | X | - |
| NIT_395 | *Leontopithecus sp.* | Male | 2013 | Niteroi | Tissue | X | X |
| NIT_488 | *Leontopithecus sp.* | Female | 2013 | Niteroi | Tissue | X | - |
| NIT_435 | *Leontopithecus sp.* | Male | 2013 | Niteroi | Tissue | X | - |
| NIT_497 | *Leontopithecus sp.* | Female | 2013 | Niteroi | Tissue | X | - |
| NIT_502 | *Leontopithecus sp.* | Male | 2013 | Niteroi | Tissue | X | - |

Mitochondrial sequences and GBS data were submitted to GenBank and SRA, respectively; and are registered under submission IDs [SUB15479739](https://submit.ncbi.nlm.nih.gov/subs/genbank/SUB15479739) and 1293468 (BioProject: 1293468), respectively.

**Figure S1**. Genetic population structure using 1,889 outlier SNPs of *Leontopithecus chrysomelas* (GHLT), *Leontopithecus rosalia* (GLT), and a captive hybrid (H) from the Primatology Center of Rio de Janeiro (CPRJ) and individuals from the allochthonous population of Niteroi (NIT), based on (A) a non-negative matrix factorization (sNMF) approach and (B) its cross-entropy (K=2); (C) principal component analysis (PCA); and (D) fixation index (F_ST_) estimates.

**Figure S2**. Alignment for the COI fragments (571 bp) obtained for *Leontopithecus chrysomelas* (GHLT), *Leontopithecus rosalia* (GLT), one captive hybrid between these species (H) from the Primatology Center of Rio de Janeiro (CPRJ), and allochthonous specimens of lion tamarins from Niteroi (Rio de Janeiro, Brazil).
